# Bevacizumab specifically decreases elevated levels of circulating KIT^+^CD11b^+^ cells and IL-10 in metastatic breast cancer patients

**DOI:** 10.18632/oncotarget.7097

**Published:** 2016-01-31

**Authors:** Sarah Cattin, Benoît Fellay, Sylvain Pradervand, Andreas Trojan, Thomas Ruhstaller, Curzio Rüegg, Gregor Fürstenberger

**Affiliations:** ^1^ Department of Medicine, Faculty of Science, University of Fribourg, CH-1700 Fribourg, Switzerland; ^2^ Central Laboratory, HFR Hôpital Cantonal, CH-1700 Fribourg, Switzerland; ^3^ Genomic Technologies Facility, Center of Integrative Genomic (CIG), University of Lausanne (UNIL), CH-1015 Lausanne, Switzerland; ^4^ OnkoZentrum Zürich, CH-8038 Zürich, Switzerland; ^5^ Breast Center, Kantonsspital St.Gallen, CH-9000 St.Gallen, Switzerland; ^6^ Tumor and Breast Center ZeTuP, CH-9006 St.Gallen, Switzerland

**Keywords:** breast cancer, angiogenesis, monocytes, KIT, IL-10

## Abstract

**Background:**

Whether bevacizumab exerts its anti-tumor properties through systemic effects beyond local inhibition of angiogenesis and how these effects can be monitored in patients, remain largely elusive. To address these questions, we investigated bone marrow-derived cells and cytokines in the peripheral blood of metastatic breast cancer patients undergoing therapy with bevacizumab.

**Methods:**

Circulating endothelial cells (CEC), circulating endothelial progenitor (CEP) and circulating CD11b^+^ cells in metastatic breast cancer patients before and during therapy with paclitaxel alone (*n* = 11) or in combination with bevacizumab (*n* = 10) were characterized using flow cytometry, real time PCR and RNASeq. Circulating factors were measured by ELISA. Aged-matched healthy donors were used as baseline controls (*n* = 12).

**Results:**

Breast cancer patients had elevated frequencies of CEC, CEP, TIE2+CD11b^+^ and KIT^+^CD11b^+^ cell subsets. CEC decreased during therapy, irrespective of bevacizumab, while TIE2^+^CD11b^+^ remained unchanged. KIT^+^CD11b^+^ cells decreased in response to paclitaxel with bevacizumab, but not paclitaxel alone. Cancer patients expressed higher mRNA levels of the M2 polarization markers CD163, ARG1 and IL-10 in CD11b^+^ cells and increased levels of the M2 cytokines IL-10 and CCL20 in plasma. M1 activation markers and cytokines were low or equally expressed in cancer patients compared to healthy donors. Chemotherapy with paclitaxel and bevacizumab, but not with paclitaxel alone, significantly decreased IL-10 mRNA in CD11b^+^ cells and IL-10 protein in plasma.

**Conclusions:**

This pilot study provides evidence of systemic immunomodulatory effects of bevacizumab and identified circulating KIT^+^CD11b^+^ cells and IL-10 as candidate biomarkers of bevacizumab activity in metastatic breast cancer patients.

## INTRODUCTION

Growing experimental evidence indicates that tumors can mobilize a diverse spectrum of bone marrow derived (BMD) and inflammatory cells and recruit them to the primary and metastatic tumor microenvironments. Upon recruitment these cells are educated to support cancer cell proliferation, survival and invasion and to induce tumor angiogenesis and matrix remodeling [1]. Mobilization and recruitment of BMD and inflammatory cells have also been associated with resistance to anti-cancer therapy, including anti-angiogenic therapy [2]. Among these cells, endothelial progenitor cells and BMD myelomonocytic cells have received particular attention in relationship to resistance to therapy [1, 3]. It has been reported that chemotherapy mobilizes endothelial progenitor cells, thereby favoring tumor angiogenesis, while concomitant anti-angiogenic therapy blunts this mobilization and enhances response to chemotherapy [4]. Blockade of monocyte/macrophage recruitment in combination with chemotherapy was shown to reduce tumor growth and improve survival of mammary tumor-bearing mice compared to chemotherapy alone [5]. Strikingly, anti-angiogenic therapy itself was reported to mobilize BMD CD11b^+^ myelomonocytic cell sub-populations with pro-angiogenic properties, thereby contributing to resistance to anti-angiogenic therapy [6]. Therefore, it has been proposed that therapy-mobilized BMD myelomonocytic cells and circulating endothelial progenitor cells (CEP) could serve as biomarkers of resistance to therapy as well as therapeutic targets to elude resistance [7]. In addition, circulating endothelial cells (CEC) were shown to be present at higher levels in the peripheral blood of cancer patients and to represent a potential biomarker for angiogenic, unstable vessels and vascular disruption by therapy [3]. The role of mobilized BMD cells, in particular CD11b^+^ cells and CEP, in predicting response and/or mediating resistance to therapy in human cancer remains undefined. We previously reported increased levels of CEC in breast cancer patients, which decreased during chemotherapy, whereas CEP increased during chemotherapy [8]. Increase in VEGFR2^+^ CEP was shown to predict relapse in non-metastatic breast cancer patients and disease progression in metastatic patients [9]. Increased levels of circulating CD11b^+^ myeloid-derived suppressor cells were reported in breast cancer and other malignancies, and were shown to correlate with cancer stage, metastatic burden and chemotherapy treatment [10–12].

Anti-VEGF therapy with bevacizumab, alone or in combination with chemotherapy, is now standard in several types of advanced/metastatic cancers [13]. At present however the mechanisms of action of bevacizumab is still matter of investigation and no biomarkers of activity or prediction of response have been identified [14]. Both subjects are clinically relevant, as only a fraction of patients initially respond to bevacizumab and most of them eventually develop resistance thereby resulting in limited overall survival benefits [15, 16].

A few studies investigated blood-circulating factors in several cancers, such as non-small-cell lung cancer (NSCLC) [17], metastatic colorectal cancer [15, 18, 19] and advanced pancreatic cancer [20] in relationship with bevacizumab therapy, and identified candidate biomarkers of response or outcome. Additional studies explored the effect of bevacizumab on circulating CEC, CEP and CD11b^+^ myelomonocytic cells and the relationship to patients' response to therapy [21–23].

A further characterization of bevacizumab effects on circulating cells might allow a more complete understanding of its mechanism of actions and possibly defining biomarkers for predicting or monitoring the response to treatment. In this pilot study we analyzed the frequency and phenotype of CEC, CEP and selected CD11b^+^ cell subsets in breast cancer patients diagnosed with metastasis before and during the first cycle of therapy with paclitaxel ± bevacizumab. To further characterize CD11b^+^ cells we performed gene expression analysis by RNASeq and validate expression of selected genes at mRNA and protein levels by real time qPCR and ELISA.

## RESULTS

### Metastatic breast cancer patients have elevated frequency of CEC and CEP

**Figure 1:**
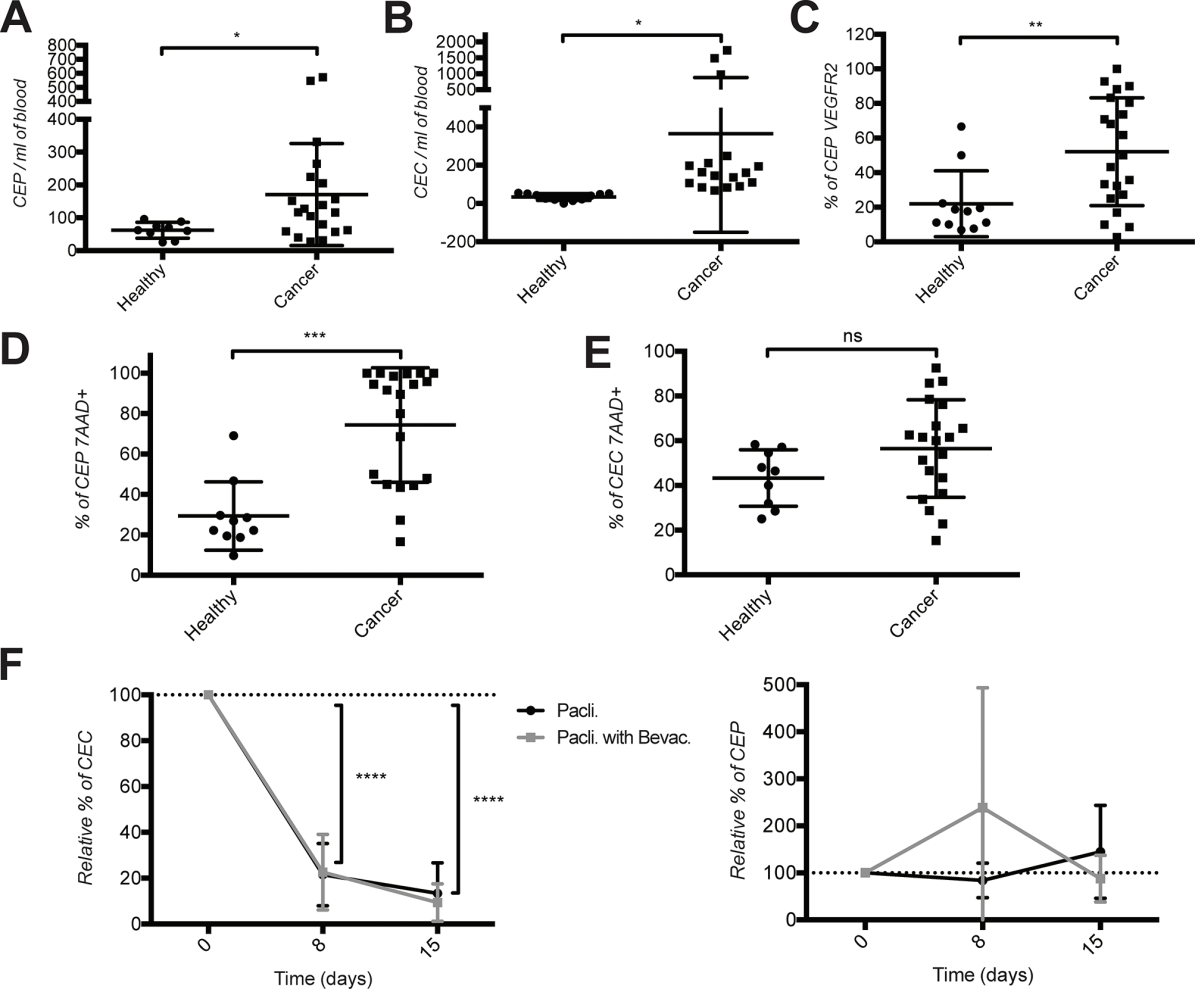
Increased frequency of CEC and CEP in the blood of metastatic breast cancer patients Quantification of (**A**) CEP, (**B**) CEC, (**C**) CEP expressing VEGFR2, (**D**) dying CEP using 7AAD staining, and (**E**) dying CEC using 7AAD staining in the blood of healthy donors and metastatic breast cancer patient before therapy start. (**F**) Quantification of CEC and CEP under chemotherapy ± bevacizumab treatments in the blood of metastatic breast cancer patients. Cell quantifications were performed by flow cytometry and data are represented as mean +/− SD.

First we quantified the frequency of CEC and CEP in the blood of 21 metastatic breast cancer (mBC) patients and 12 age-matched healthy donors by cell surface staining and flow cytometry following the protocol by Bertolini *et al.* [24, 25] (Table 1). CEC were defined as CD45^−^DNA^+^CD146^+^CD31^+^ cells and CEP as CD45^−^DNA^+^CD133^+^CD34^+^ cells. Compared to healthy donors, breast cancer patients had elevated levels of CEC and CEP (Figure 1A–1B). CEPs in mBC patients were also significantly more positive for VEGFR2 expression compared to healthy donors (Figure 1C). Surprisingly the fraction of dying CEP, but not CEC, determined using 7AAD^+^ permeability staining, was significantly higher in mBC patients (Figure 1D–1E).

**Table 1:** Clinical data of metastatic breast cancer patients

Patient Nbr	Age	Treatment	ER (+/−)	PR (+/−)	HER2 (+/−)	PT subtype	PT grade	Metastasis (time after PT)	Metastasis location	Biphosphonate/anti-RANK-L (after therapy start)	Hormonal treatment
1	78	pacli.	+	+	−	Luminal - A	3	9 years	Lymph node, Liver	-	-
2	58	pacli.	+	+	−	Luminal - A	3	simultaneous	Lymph node, Bone	Bondronat (day -7)	letrozole
3	78	pacli.	+	+	−	Luminal - A	1	16 years	Bone	Bondronat (day 8)	letrozole + fulvestrant
4	69	pacli.	+	+	−	Luminal - A	3	4.5 years	Lymph node	-	tamoxifen + arimidex
5	55	pacli.	−	−	−	Triple neg.	3	simultaneous	Lymph node, Liver, Bone	Zometa (day -14)	-
6	73	pacli.	+	−	−	Luminal - A	ND	4.5 years	Bone	Denosumab (day -3)	-
7	72	pacli.	+	−	−	Luminal - A	2	simultaneous	Lymph node, Bone, Lung	Bondronat (day 1)	tamoxifen
8	64	pacli.	+	−	+	Luminal - B	2	simultaneous	Bone, Liver, Peritoneum	-	tamoxifen
9	53	pacli.	+	−	−	Luminal - A	3	10.5 years	Lymph node, Bone, Lung	Bondronat (day 1)	exemestan + everolimus
10	49	pacli.	+	+	+	Luminal - B	3	10.5 years	Lymph node, Lung	-	fulvestrant
11	76	pacli.	−	−	+	HER2+	3	10 years	Bone, Lung, Liver, Brain	Zometa (day 8)	-
12	67	pacli. + bevac.	+	+	−	Luminal - A	ND	simultaneous	Bone, Lung	Pamidronat (day -3)	-
13	48	pacli. + bevac.	+	+	−	Luminal - A	ND	3.5 years	Bone, Liver	Bondronat (day 8)	-
14	45	pacli. + bevac.	−	−	−	Triple neg.	2	simultaneous	Lymph node, Bone	Zometa (day 8)	tamoxifen
15	39	pacli. + bevac.	+	+	−	Luminal - A	3	6 years	Bone, Lung	Denosumab (day 1)	letrozole
16	47	pacli. + bevac.	+	−	−	Luminal - A	3	6 months	Bone	-	exemestan
17	45	pacli. + bevac.	−	−	−	Triple neg.	3	2 years	Lymph node, Liver, Bone	-	-
18	65	pacli. + bevac.	+	+	−	Luminal - A	2	simultaneous	Lymph node, Bone, Breast	-	-
19	77	pacli. + bevac.	−	−	−	Triple neg.	ND	1.5 years	Lymph node, Breast, Skin	-	-
20	49	pacli. + bevac.	−	−	−	Triple neg.	3	1 year	Bone	Denosumab (day -3)	-
21	49	pacli. + bevac.	+	−	−	Luminal - A	3	simultaneous	Lymph node, Liver, Bone	-	-

Patient's demographics, treatments, tumor type and grading, metastasis. ER: estrogen receptor expression; PR: progesterone receptor expression; HER2: overexpression of HER-2/neu; PT: primary tumor.

### Bevacizumab treatment has no additional effect on CEC and CEP over paclitaxel chemotherapy

To monitor the effect induced by paclitaxel ± bevacizumab on circulating CEC and CEP, we analyzed blood from 11 patients upon breast metastasis detection treated first line with one cycle of paclitaxel chemotherapy alone and from 10 patients treated with paclitaxel in combination with bevacizumab. Blood was taken before therapy start (day 0) and at days 8 and 15 immediately before the administration of the treatment. Under chemotherapy the frequency of CEC dropped by over 80% regardless of the presence of bevacizumab (Figure 1F, left panel). The number of CEP doubled at day 8 of therapy with paclitaxel and bevacizumab (although the difference is not statistically significant due to large variability in the values) and returned to normal at day 15. Therapy with paclitaxel alone had no effect.

These data demonstrate the presence of elevated levels of blood circulating CEC and CEP in patients with mBC, and that bevacizumab treatment has no effect on their frequency in addition to chemotherapy at the investigated time points.

### Increased frequency of circulating TIE2^+^CD11b^+^ and KIT^+^CD11b^+^ cells in mBC patients

**Figure 2:**
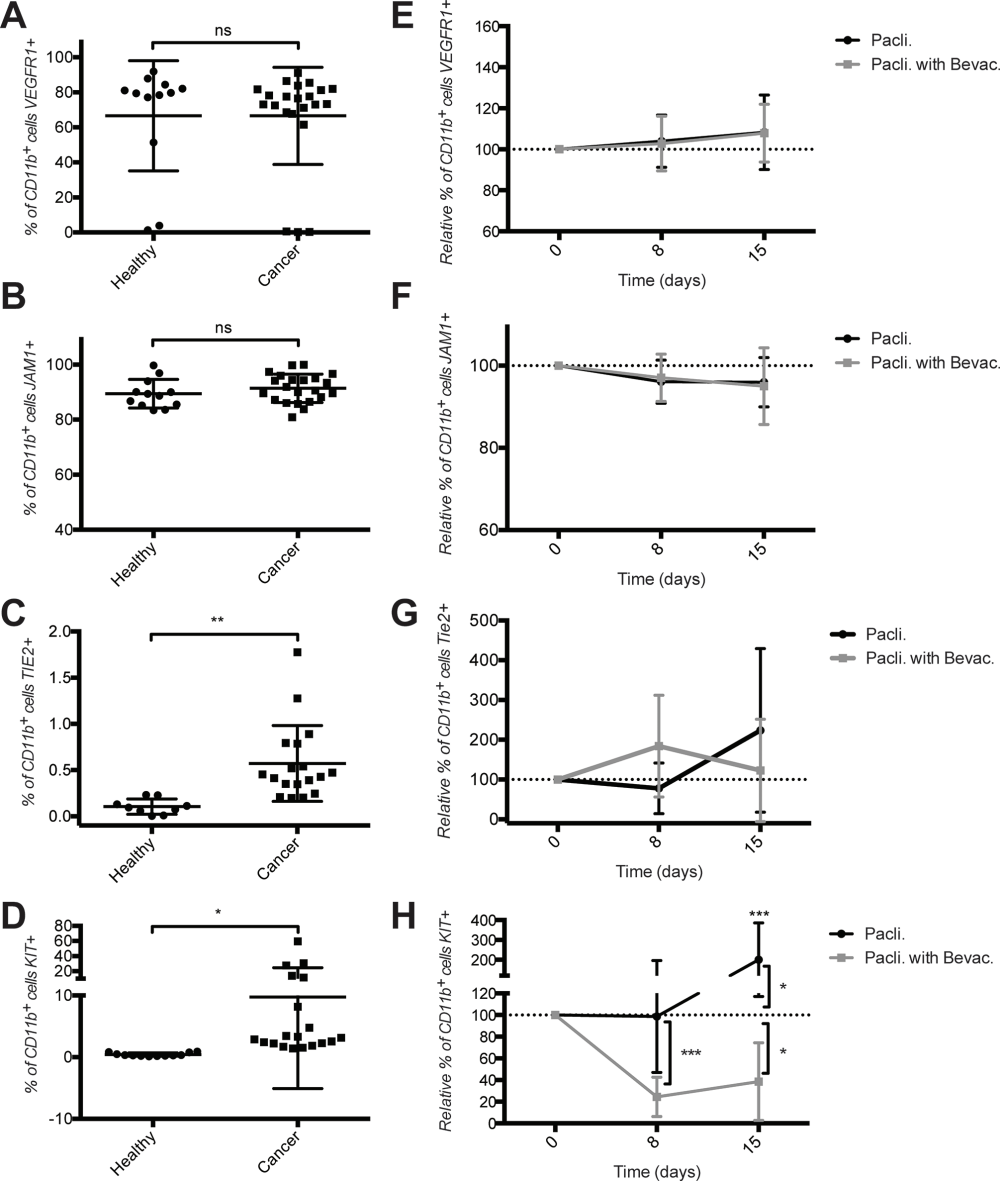
Increased frequency of TIE2^+^CD11b^+^ and KIT^+^CD11b^+^ cells in the blood of metastatic breast cancer patients and decreased frequency of KIT^+^CD11b^+^ cells by bevacizumab therapy Quantification of CD11b^+^ cells expressing (**A**) VEGFR1, (**B**) JAM1, (**C**) TIE2 and (**D**) KIT in the blood of healthy donors and breast cancer patients before therapy start. Quantification under both chemotherapy treatments of CD11b+ cells expressing (**E**) VEGFR1, (**F**) JAM1, (G) TIE2 and (**H**) KIT in the blood of metastatic breast cancer patients. Cell quantifications were performed by flow cytometry and data are represented as mean +/− SD.

We previously observed elevated levels of VEGFR1^+^CD11b^+^ [26], KIT^+^CD11b^+^ [27] and JAM1^+^CD11b^+^ (unpublished data) in murine models of breast cancer and TIE2^+^CD11b^+^ cells in mBC cancer patients [28]. We therefore hypothesized that the frequency of CD11b^+^ cells and subsets thereof could potentially reflect the presence of a tumor and possibly patients' response to the treatment. To test this hypothesis we used flow cytometry to measure CD11b^+^ cell subsets in healthy donors and in patients with mBC before and during therapy. Frequencies of circulating TIE2^+^CD11b^+^ and KIT^+^CD11b^+^ cells, but not VEGFR1^+^CD11b^+^ or JAM1^+^CD11b^+^ cells were significantly elevated in mBC patients compared to healthy donors (Figure 2A–2D). JAM1^+^ or VEGFR1^+^ cells represented a large fraction of the CD11b^+^ cell population (approx. 90% and 70%, respectively). In contrast, the relative frequency of TIE2^+^ cells within the CD11b^+^ cells was very low (< 1% in average), and that of KIT^+^ cells was spread over a large spectrum, ranging from 2–5% to 60% of the CD11b^+^ cells. Importantly, KIT^+^CD11b^+^ cells were extremely rare in healthy donors (0.3% of CD11b^+^ cells).

As we observed a large variation of KIT protein expression at the cell surface of mBC patients, we tested whether KIT mRNA expression in CD11b^+^ blood cells was more homogeneous. CD11b^+^ cells were isolated from fresh blood of mBC patients and healthy donors by MACS technology and analyzed for KIT mRNA levels by qPCR. Purity of isolated CD11b^+^ cells was > 98% (Supplementary Figure 1). Surprisingly results revealed that KIT mRNA expression levels were very low in mBC patients and indistinguishable from healthy donors (Supplementary Figure 2).

### Bevacizumab reduces the frequency of circulating KIT^+^CD11b^+^ in paclitaxel-treated patients

Next we measured the effect of therapy on CD11b^+^ cell subsets. Therapy with ± bevacizumab did not significantly alter the frequency of VEGFR1^+^CD11b^+^, JAM1^+^CD11b^+^ or TIE2^+^CD11b^+^ cells (Figure 2E–2G). In contrast, therapy with paclitaxel caused a significant 2-fold increase in the frequency of circulating KIT^+^CD11b^+^ cells at day 15, while addition of bevacizumab resulted in a significant drop (60–80%) at days 8 and 15 (Figure 2H).

From these experiments we concluded that circulating TIE2^+^CD11b^+^ and KIT^+^CD11b^+^ cells are enriched in mBC, and bevacizumab decreases KIT^+^CD11b^+^, but not TIE2^+^CD11b^+^ cells.

### Circulating CD11b^+^ cells in mBC patients have a distinctive gene expression profile compared to healthy donors

**Figure 3:**
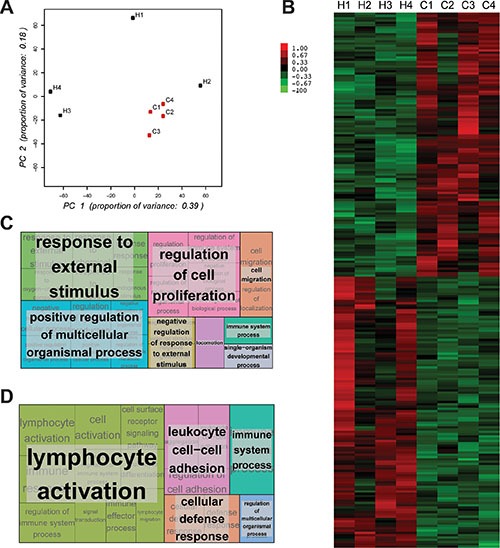
CD11b^+^ myelomonocytic cells from metastatic breast cancer patients and healthy donors show different expression profiles (**A**) PCA plot representing differential clustering of cancer patients (C1-4) and healthy donors (H1-4) based on mRNA expression profiles of CD11b^+^ cells. (**B**) Self-organizing heat-map of the top 100 genes with greatest variability across all samples, showing different expression profiles in CD11b^+^ cells from cancer patients compared to healthy donors. (**C**) Gene ontology analysis showing the most up-regulated biological processes comparing cancer patients and healthy donors. (**D**) Gene ontology analysis showing the most down-regulated biological processes comparing cancer patients and healthy donors.

To further characterize CD11b^+^ cells in mBC patients we performed RNASeq analysis on CD11b^+^ cells isolated from 4 healthy donors and 4 cancer patients. Principal Component Analysis (PCA) revealed that the gene expression profiles of CD11b^+^ cells derived from mBC patients clustered together while those from CD11b^+^ cells derived from healthy donors were dispersed (Figure 3A). This result suggests that in healthy conditions CD11b^+^ cells have rather heterogeneous gene expression across individuals, while in cancer a re-programming takes place resulting in a more homogenous gene expression across patients. Self-organizing heat-maps confirmed that CD11b^+^ cells from cancer patients and healthy donors display different gene expression profiles (Figure 3B). Interestingly, among the most differentially expressed genes, we observed that CD11b^+^ cells from mBC patients expressed genes of M2 monocytes' activation state (i.e. IL-10 and CCL20) (Supplementary Table 1). Gene ontology analysis of the differentially expressed genes (Figure 3C–3D and Supplementary Tables 2–3) reveled that expression of immune response genes was reduced in mBC, consistent with a M2 tumor-induced CD11b^+^ cells polarization.

### CD11b^+^ monocytes in mBC patient display a M2 activation phenotype

**Figure 4:**
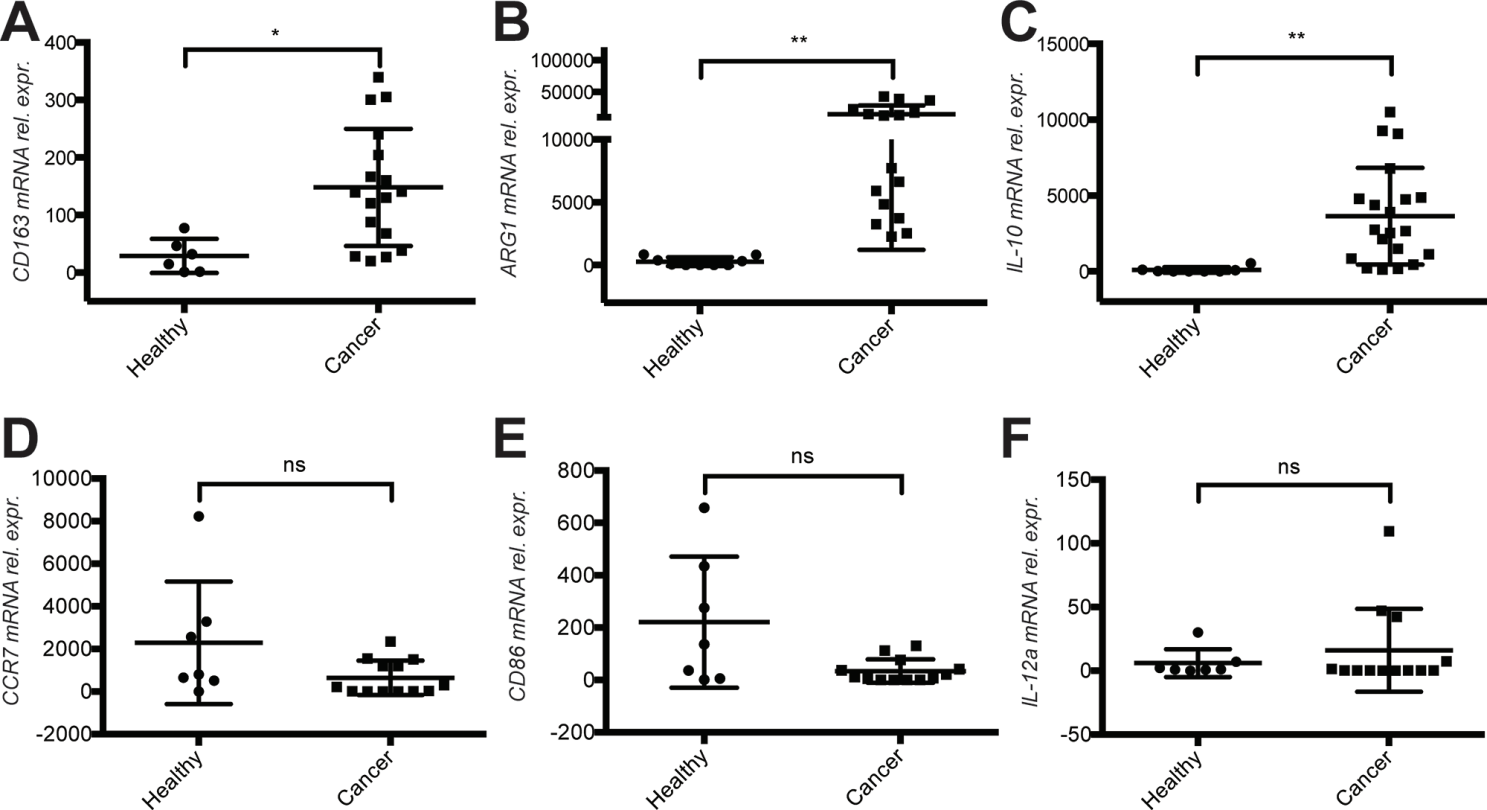
Evidence for a M2 activation phenotype of CD11b^+^ cells in the blood of metastatic breast cancer patients (**A**) Quantification of the mRNA expression levels of M2 markers CD163, (**B**) ARG1 and (**C**) IL-10 in CD11b+ cells derived from the blood of healthy donors compare to cancer patients before therapy start. (**D**) Quantification of mRNA expression levels of M1 markers CCR7, (**E**) CD86 and (**F**) IL-12α at mRNA level in CD11b^+^ cells of the same healthy donors and cancer patients. Analysis was performed by real time qPCR. All data are represented as mean +/− SD.

To strengthen this observation we monitored the expression of additional transcripts associated with M2 activation states in CD11b^+^ cells derived from healthy donors and mBC patients. IL-10, ARG1 and CD163, typical M2 activation markers, were highly up regulated in mBC patient (Figure 4A–4C). In contrast, the M1 activation markers CCR7, CD86 and IL-12a were expressed at lower levels, albeit not significantly (Figure 4D–4F). We also validated the increased expression of fibronectin mRNA in CD11b^+^ cells of mBC patients (Supplementary Table 1 and Supplementary Figure 3).

From these results we concluded that circulating CD11b^+^ cells in mBC have a different gene expression profile compared to healthy donors, and express genes associated with a M2, but not M1 activation.

### Increased levels of the circulating M2 cytokines IL-10 and CCL20 in mBC patients

**Figure 5:**
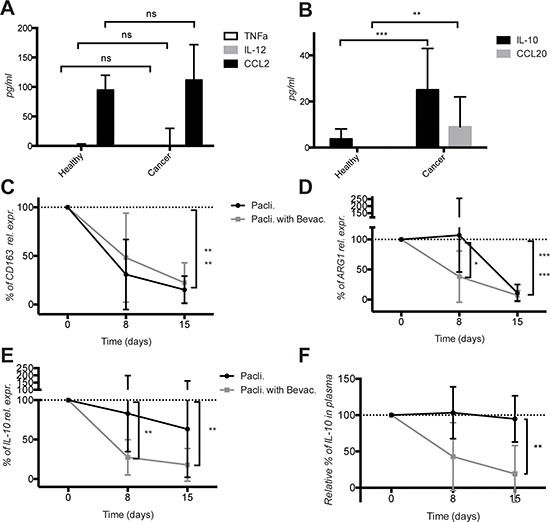
Increased expression of the M2 cytokines IL-10 and CCL20 in the plasma of metastatic breast cancer patients and decrease of IL-10 levels by bevacizumab (**A**) Quantification of M1 cytokines TNFα, IL-12p70 and CCL2 in plasma of healthy donors and cancer patients before therapy start. (**B**) Quantification of M2 cytokines IL-10 and CCL20 in the plasma of the same healthy donors and cancer patients before therapy start. (**C**) Quantification of CD163, (**D**) ARG1 and (**E**) IL-10 mRNA expression variation in CD11b^+^ cells of cancer patient during therapy with paclitaxel ± bevacizumab using real time qPCR. (**F**) Quantification of IL-10 in the plasma of cancer patients during therapy with paclitaxel ± bevacizumab. Cytokines were measured by ELISA. All data are represented as mean +/− SD.

To collect additional evidence that the M2 activation in mBC was a systemic event, we measured selected M1 and M2 cytokines in the plasma of healthy donors and mBC patients. The M1 cytokines TNFα and IL-12 were below detection levels, while CCL2 was detectable, but no differences were observed (Figure 5A). In contrast, the level of the M2 cytokines IL-10 and CCL20 was significantly higher in mBC patients compared to healthy donors (Figure 5B). These results corroborate the mRNA expression data consistent with a M2 activation state of circulating CD11b^+^ cells in mBC patients.

### Bevacizumab diminishes IL-10 mRNA expression in CD11b^+^ cells and protein level in plasma

Next we monitored the effect of therapy on the elevated M2 markers. Due to limited number of CD11b^+^ cells and derived mRNA recovered form treated patients, we focused the analysis on a few genes only. The mRNA expression level of CD163 and ARG1 clearly decreased in cancer patients under chemotherapy regardless of the presence of bevacizumab, although bevacizumab accelerated the decrease of ARG1 (Figure 5C–5D). Importantly, we observed a significant decrease of IL- 10 both at mRNA level in CD11b^+^ cells and at protein levels in the plasma of patients treated with bevacizumab in addition to paclitaxel (Figure 5E and 5F). Furthermore we observed a stronger increase in CD86 mRNA level in CD11b^+^ cells and CCL2 protein level (both reflecting M1 activation state) in plasma of mBC patients treated with paclitaxel + bevacizumab compared to patients treated with paclitaxel only. However due to a greater variability and the small number of patients, differences were not statistically significant (Supplementary Figure 4).

These results demonstrate that mBC patients have M2-activated circulating CD11b^+^ cells and elevated levels of M2 cytokines in plasma. Bevacizumab addition to chemotherapy causes a significant decrease of IL-10 mRNA levels in CD11b^+^ cells and IL-10 protein levels in plasma.

## DISCUSSION

The mode of action of bevacizumab is not yet fully understood. Beyond a rather transient anti-angiogenic effect, a possible more complex, systemic effect has been proposed [29, 30]. Additionally, immunomodulatory effects of anti-angiogenic therapies have been discussed [31]. To collect evidence supporting systemic, immunomodulatory effects of bevacizumab, and to identify candidate biomarkers to predict or monitor response to treatment, we investigated the effect of bevacizumab in addition to paclitaxel, on CEC, CEP and CD11b^+^ cell subsets in the blood of mBC patients.

Beyond the fact that CEC and CEP are present at higher frequency in the blood of mBC patients compared to healthy donors and that paclitaxel therapy decreases CEC independently of bevacizumab, no relationship between anti-angiogenic treatment and CEP behavior are observed. We couldn't validate the mobilization of CEP by paclitaxel and the inhibitory effect by bevacizumab previously reported in preclinical and clinical studies [4, 8]. Rather we observed a transient (at day 8) but statistically non-significant increase of CEP in patients treated with paclitaxel and bevacizumab compared to paclitaxel treatment alone. The discrepancy of the observation on CEP may be due to the different time points analyzed (a few hours vs. 8 and 15 days after therapy start) and to differences in the definition and analysis of CEP across these studies.

In contrast, we observed a KIT^+^CD11b^+^ population present only in the peripheral blood of cancer patients and whose frequency decreased significantly under therapy with paclitaxel and bevacizumab compared to paclitaxel only. This effect is specific, as three additional subpopulations of CD11b^+^ cells (VEGFR1^+^, JAM1^+^, TIE2^+^) were not affected. We have previously reported that circulating KIT^+^CD11b^+^ cells promote metastasis in the 4T1 murine model of breast cancer relapse after radiotherapy [27]. Little was known, however, about the clinical and biological significance of KIT^+^CD11b^+^ cells in patients. Here we demonstrate that such cells circulate in the blood of patients with metastatic breast cancer but are virtually absent in healthy individuals. This observation raises several questions. A critical one is whether KIT^+^CD11b^+^ cells might be a potential biomarker paralleling activity of, or predicting response to, bevacizumab treatment. The broad distribution of KIT^+^CD11b^+^ frequency suggests that patients may be heterogeneous in term of KIT^+^CD11b^+^ mobilization or recruitment. The potential therapeutic significance of this observation is open at this point, and further studies including more patients and monitoring clinical outcome are necessary to address it. The origin, role and fate of these cells in cancer progression are also intriguing. The discrepancy observed between levels of KIT protein and mRNA is consistent with immature myeloid-derived cells shutting down KIT expression upon mobilization. These cells, once in the blood circulation, may be on the way to differentiation and therefore no longer express KIT mRNA, while still carry KIT protein at the cell surface. In the 4T1 model we reported that mobilized Kit^+^CD11b^+^ cells rapidly lose KIT surface expression upon adoptive transfer [27].

The gene expression profiling of CD11b^+^ cells demonstrated significant differences between cancer patients and healthy donors. Strikingly, expression profiles of CD11b^+^ cells from cancer patients were more homogeneous compared to those of healthy donors. This is consistent with the functional heterogeneity CD11b^+^ cells in healthy individuals reflecting physiological individualistic behaviors vs. a tumor-driven CD11b^+^ reprogramming resulting in more homogenous gene expression profiles. Consistent with this notion, we observed the expression of transcripts for M2 activation markers in CD11b^+^ cells of cancer patients in comparison to CD11b^+^ cells from healthy controls. These observations are corroborated by higher levels of IL-10 and CCL20, two M2-associated cytokines, in the plasma of cancer patients. IL-10 is a well-known immune suppressive cytokine produced during cancer progression [32]. CCL20 is involved in promoting tumor growth, migration and EMT transition in breast cancer *in vitro* models [33] and it also plays a role in recruiting immature dendritic cells and in tumor-promoting Tregs in other cancer types [34]. We also found elevated fibronectin (FN1) transcript in CD11b^+^ cells of cancer patients. Interestingly, fibronectin deposition is observed in the metastatic niche in preclinical models in associated in with recruitment of CD11b^+^ cells [35] and with cancer progression, including of the breast, in human [36].

Unfortunately it was not possible to monitor the therapy effect on the M2 phenotype by RNASeq due to insufficient amounts and low quality material. However, we were able to measure selected transcripts for M1 and M2 markers in CD11b^+^ cells and M1 and M2 cytokines in the plasma of patients under therapy. Most of them didn't change or were below detection level (data not show). Nevertheless we observed significant and specific decrease in the M2 markers ARG1 at mRNA level (albeit ARG1 was only significant at day 8) and IL-10 cytokine secretion under bevacizumab therapy, and a consistent, but not significant, trend in increased M1 markers CD86 and CCL2 cytokine production. These results suggest that bevacizumab could prompt an immune switch from a pro-tumoral M2 activity toward an anti-tumoral M1 activity. Tumor-induced M2 polarization and cytokine production may reach beyond local reprogramming occurring in the tumor microenvironment and may have systemic relevance [28, 37].

These observations may have practical implications to breast cancer diagnosis, treatment and monitoring. Circulating KIT^+^CD11b^+^ cells and IL-10 levels (or more generally M2/M1 marker ratio) could serve as potential biomarkers to follow patient's response to bevacizumab treatment. This might be particularly relevant in conditions where bevacizumab effects may be restricted to a subset of the patients, as it appears to occur in metastatic breast cancer. Their modulation is consistent with an immunostimulatory effect of bevacizumab. Interestingly, preclinical and clinical evidence suggest that response to chemotherapy involves the activation of the immune system and induction of an anti-tumor immune response [38, 39]. Thus bevacizumab, in addition to its anti-angiogenic effects, might provide therapeutic benefits by reversing the M2-associated state of immunosuppression. Larger studies are needed to test this hypothesis.

Most patients received bisphosphonates (or an anti-RANK-L antibody) as part of their therapies due to bone metastasis (Table 1), but no correlations were observed between marker levels and bisphosphonate (or anti-RANK-L) treatment (Supplementary Figures 5 and 6), except a transient increased mobilization of CEP at day 8 of therapy with paclitaxel and bevacizumab. Bisphosphonates have been involved in modulation of bone-resident macrophage activity including reprogramming from tumorigenic M2 status to an anti-tumoral M1 status [40]. Bisphosphonate effects on circulating CD11b^+^ cells cannot be confirmed by our data (data not show). Also, the limited number of patients did not allow drawing an association between a particular subtype of breast cancer and circulating cells (Supplementary Figure 7). In addition, some patients also received in parallel an endocrine therapy based on their cancer subtype (Table 1). These treatments also did not have any effect on our cells and markers levels (data not show). The fact that some patients had primary tumor surgery more then 6 months before treatment start (Table 1) also did not have any significant impact on the observed changes on CEC, CEP, KIT^+^ CD11b^+^ cells and M2 markers (data not show). It should be emphasized that considering the small number of patients analyzed, these lack of correlations do not exclude that some correlations might be observed in larger patients populations.

In summary, we demonstrate that metastatic breast cancer patients have an increased frequency of circulating KIT^+^CD11b^+^ cells, M2-polarized CD11b^+^ cells and elevated levels of the M2 cytokine IL-10 and CCL20. Compared to paclitaxel monotherapy only, co-treatment with bevacizumab significantly decreased KIT^+^CD11b^+^ cells and IL-10. These results warrant the further investigation of changes in circulating KIT^+^CD11b^+^ cells and IL-10 levels as candidate biomarkers of bevacizumab activity in metastatic breast cancer and possibly other cancer types. They also provide original evidence for a systemic immunomodulatory effect of bevacizumab. We are planning a larger study to address these questions.

## MATERIALS AND METHODS

### Clinical data and material

The study was approved by the regional ethic boards of St. Gallen and Zurich, Switzerland. We studied 21 female patients (Table 1) with metastatic breast cancer who received first line treatment of palliative chemotherapy with either paclitaxel on day 1, 8 and 15 of a 28 days cycle, or paclitaxel (same schedule) in combination with bevacizumab on day 1 and 15. From these patients, blood samples were collected before administration of the first therapy at day 0, and before therapy on day 8 and 15. All donors gave written informed consent before study entry. Patients were recruited in St. Gallen Kantonspital, Zürich Oncozentrum and Tumor- und Brustzentrum in Chur upon diagnosis of breast cancer metastasis and before starting any new treatment. Samples from 12 age-matched healthy females were analyzed accordingly. Age mean for cancer patient was 56.5 years and for healthy donors was 58 years old (all patients were between 39 and 78 years old). Healthy donors were recruited at HFR Hôpital Cantonal Fribourgeois in parallel to breast cancer patient recruitment, based on the following criteria: age-matched relative to breast cancer patient, no surgical intervention in the past 10 years, no regular medications in the last 6 months, no previous cancer diagnosis, no chronic diseases and normal blood analyses at time of recruitment.

### Blood processing

Blood was collected using BD Vacutainer^®^ Blood Collection EDTA Tubes (Becton Dickinson, Franklin Lakes, NJ, USA) following manufacturer's instructions. All following analysis were performed without 24 h after blood collection to minimize cell death. Plasma and total white blood cells were isolated using BD Vacutainer^®^ CPT^™^ Cell Preparation Tube (Becton Dickinson) with Sodium Heparin following manufacturer's instructions of use. Plasma fraction was immediately frozen at −80°C. CD11b^+^ cells were isolated from total blood using MACS^®^ separation technique, following manufacturer's instructions. MS columns and anti-human/mouse CD11b micro-beads were used. All reagents were from Miltenyi Biotec (Bergisch Gladbach, Germany). Isolated CD11b^+^ cells were immediately lysed in RNA stable RLT lysis buffer (Qiagen, Venlo, Netherlands) and stored at −80°C.

### Flow cytometry

Whole blood staining's were performed within 24 hours after blood collection. White blood cells were counted using Cell-Dyn Sapphire Hematology System (Abbott Diagnostics, Chicago, IL, USA). For CEC and CEP staining 5 millions cells per tube were used, while for monocyte and isotype staining's 1 million cells was used. Directly labeled antibodies were added on total blood and incubated for 20 minutes at 4°C, followed by 10 minutes red-blood-cells lysis (Bühlmann Laboratories, Schönenbuch, Switzerland) and washing using cold PBS.

All anti-human antibodies were used at the concentration give in manufacturer's instructions of use: anti-CD45-V450, anti-CD146-Pe, anti-CD31-AlexaFluo 647, anti-CD34-PeCy7, anti-VEGFR2-Pe, anti-CD11b-V450, anti-CD64-FITC, anti-JAM1-Pe, anti-TIE2-AlexaFluo 647, anti-KIT-PeCy7, anti-CD195-Pe, mouse IgG2a-AlexaFluor 647, mouse IgG1-Pe, mouse IgG1-PeCy7, mouse IgG1-APC, mouse IgG2a-Pe, 7AAD (all from Becton Dickinson), Syto16 (Life Technologies, Carlsbad, CA, USA), anti-CD133-APC (Miltenyi), anti-VEGFR1-APC (R & D Biosystems, Minneapolis, MN, USA).

BD FACSCanto II (Becton Dickinson) instrument was used to perform experiments and FlowJo 9.8.3 (Treestar Inc., Ashland, OR, USA) software was used to analyze all data.

### ELISA

For TNF-α, IL-12p70, IL-10, CCL20 and CCL2 quantification in plasma ELISA MAX Deluxe assays from Biolegend (San Diego, CA, USA) was used following manufacturer's instructions. Each sample was run in triplicate and absorption was measured at 450 nm (VICTOR X3 Reader, PerkinElmer, Waltham, MA, USA).

### Real-time qPCR

Total RNA from CD11b^+^ isolated cells was extracted using the RNeasy Mini kit from Qiagen. The quality and quantity of all RNA samples were examined by Agilent 2100 Bioanalyzer (Agilent Technologies, Santa Clara, CA, USA) and NanoDrop (Witec AG, Luzern, Switzerland). Total RNA was retro-transcribed using Ovation Nugen RNASeq System v2 kit following manufacturer's instructions (Nugen, San Carlos, CA, USA) using 15 ng of total RNA. cDNA was subjected to amplification by real time qPCR with the StepOne SYBR System (Life Technologies) using the following primer pairs (Eurofins Genomics, Huntsville, AL, USA) at the indicated hybridization temperatures: GAPDH 58°C (Fw-TCTTCTTTTGCGTCGCCAGC, Rev-GATT TTGGAGGGATCTCGCTCCT), CCR7 58°C (Fw- ATG GACCTGGGGAAACCAAT, Rev- TGATAGGGAGGAA CCAGGCT), CD86 58°C (Fw- TCATTGCCGAGGAA GGCTTG, Rev- CTCCATTGTGTTGGTTCCACATT), IL-12a 60°C (Fw- TTCCCATGCCTTCACCACTC, Rev- ACT CCCATTAGTTATGAAAGAGGTC), CD163 58°C (Fw- GCGGCTTGCAGTTTCCTCAA, Rev- CTCAGAATG GCCTCCTTTTCCA), ARG1 58°C (Fw- GGAGTC ATCTGGGTGGATGC, Rev- CTGGCACATCGGGAATC TTTC), IL-10 58°C (Fw- CGAGATGCCTTCAGCA GAGT, Rev- AATCGATGACAGCGCCGTAG), KIT 57°C (Fw- GATTATCCCAAGTCTGAGAATGAA, Rev- CGTC AGAATTGGACACTAGGA). FN1 52°C (Fw- ACTTC GACAGGACCACTTGA, Rev- TCAAATTGGAGATT CATGGGA). Real-time PCR data were then analyzed using the comparative Ct method [41].

### Gene expression analysis by RNASeq

RNASeq libraries preparation, data sequencing and bioinformatics analysis was performed following previously published methodology [42]. But purity-filtered reads were adapted and quality trimmed wit Cutadapt (v.1.3), filtered for low complexity with PRINSEQ (v.0.20.3) and aligned against *Homo sapiens.GRCh37.75* genome using TopHat (v.2.0.9) [43, 44]. Quality of the RNASeq data alignment was assessed using RSeQC (v.2.3.7) [45]. The number of read counts per gene locus was summarized with htseq-count (v.0.6.1) using *Homo sapiens.GRCh37.75* gene annotation [46]. Data normalization and differential expression analysis were performed in R (v.3.1.1), using Bioconductor packages DESeq2 (v.1.6.2) [47]. The 15996 genes with at least 1 count per million in at least one sample were kept for statistical analysis. Gene Ontology (GO) analysis was performed using Gorilla [48] and representations were generated using REVIGO [49]. Significant enriched GO terms were identified after Bonferroni correction (*P* value < 4.13e-06). The complete data set is publicly available at GEO through the accession number GSE70404.

### Statistical analysis

Acquired data were analyzed and graphics were generated using Prism Software (GraphPad, La Jolla, CA, USA). Statistical comparisons were performed by two-way ANOVA with Bonferroni post-test. Highest and lowest values from each group were excluded. Results were considered to be significantly from *p* < 0.05. In the figures the various *p* values thresholds are presented as follow: ≤ 0.05 =*, ≤ 0.01 =**, ≤ 0.001 =***, ≤ 0.0001 =****.

## SUPPLEMENTARY MATERIALS FIGURES AND TABLES








