# Supplementary material for: Bevacizumab specifically decreases elevated levels of circulating KIT+CD11b+ cells and IL-10 in metastatic breast cancer patients
Source: Oncotarget. 2016 Jan 31;7(10):11137–50. doi: 10.18632/oncotarget.7097 (PMC4905463; doi:10.18632/oncotarget.7097)
Supplement: Supplementary file 1 [file oncotarget-07-11137-s001.pdf]

Bevacizumab specifically decreases elevated levels of circulating KIT⁺CD11b⁺ cells and IL-10 in metastatic breast cancer patients

Supplementary Materials

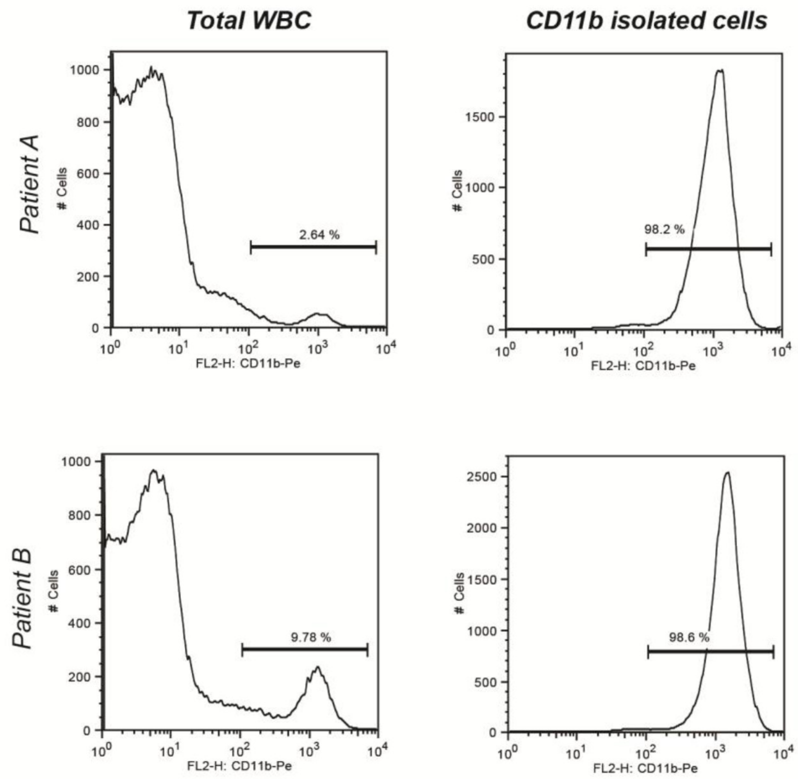

Supplementary Figure S1: High purity of MACS-isolated CD11b⁺ cells. Flow Cytometry analysis of total white blood cells isolated from two cancer patients and cells isolated thereof using MACS CD11b beads. Cells were stained with anti-CD11b-PE antibodies and analyzed by flow cytometry.

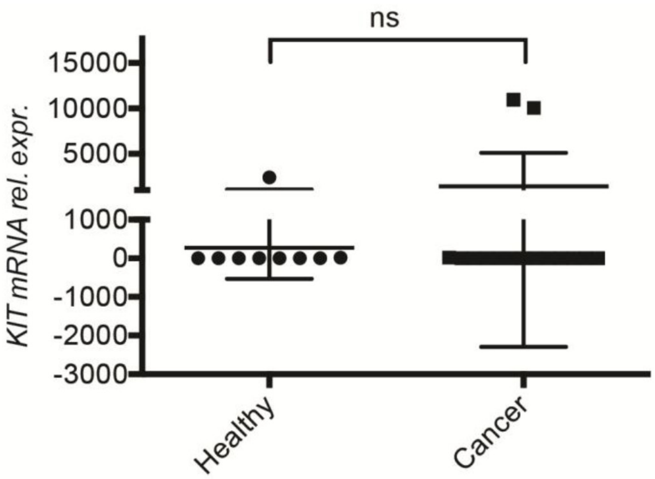

Supplementary Figure S2: Altered KIT protein expression in healthy subjects vs. cancer patients is not reflected at mRNA level. Quantification of KIT mRNA expression in CD11b⁺ cells at mRNA level using real time qPCR. Data are represented as mean +/− SD.

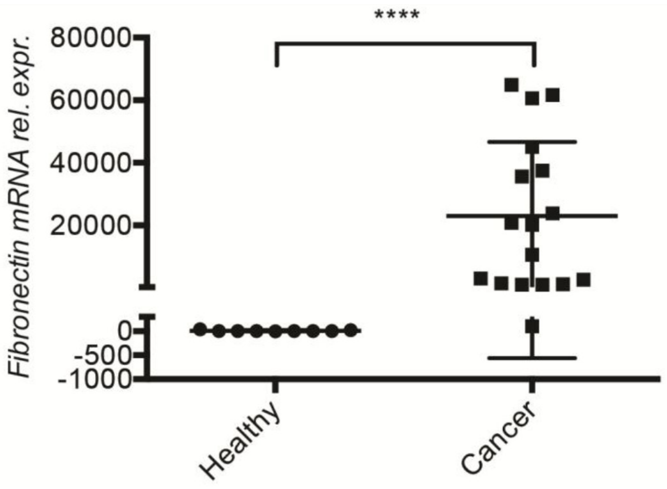

Supplementary Figure S3: Cancer patients express higher levels of fibronectin mRNA compared to healthy subjects. Quantification of fibronectin mRNA expression in CD11b$^+$ cells from healthy donors vs. breast cancer patients using real time qPCR. Data are represented as mean +/− SD.

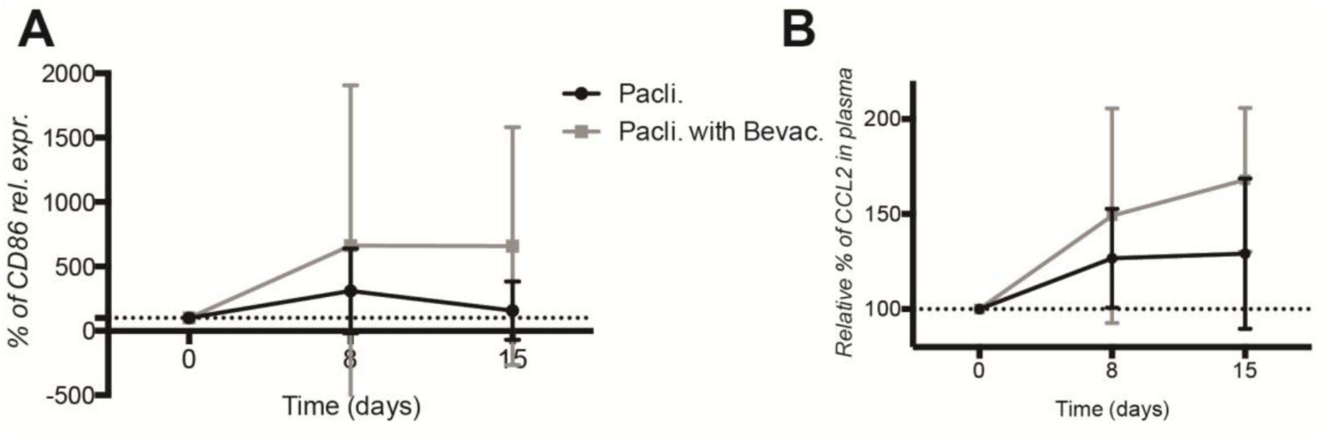

Supplementary Figure S4: M1 polarization markers tend to increase upon treatment with bevacizumab. (**A**) Quantification of the CD86 mRNA expression level in CD11b$^+$ cells from patients during therapy with paclitaxel ± bevacizumab using real time qPCR. (**B**) Quantification of CCL2 cytokine in the plasma of cancer patients during therapy with paclitaxel ± bevacizumab using ELISA. All data are represented as mean +/− SD.

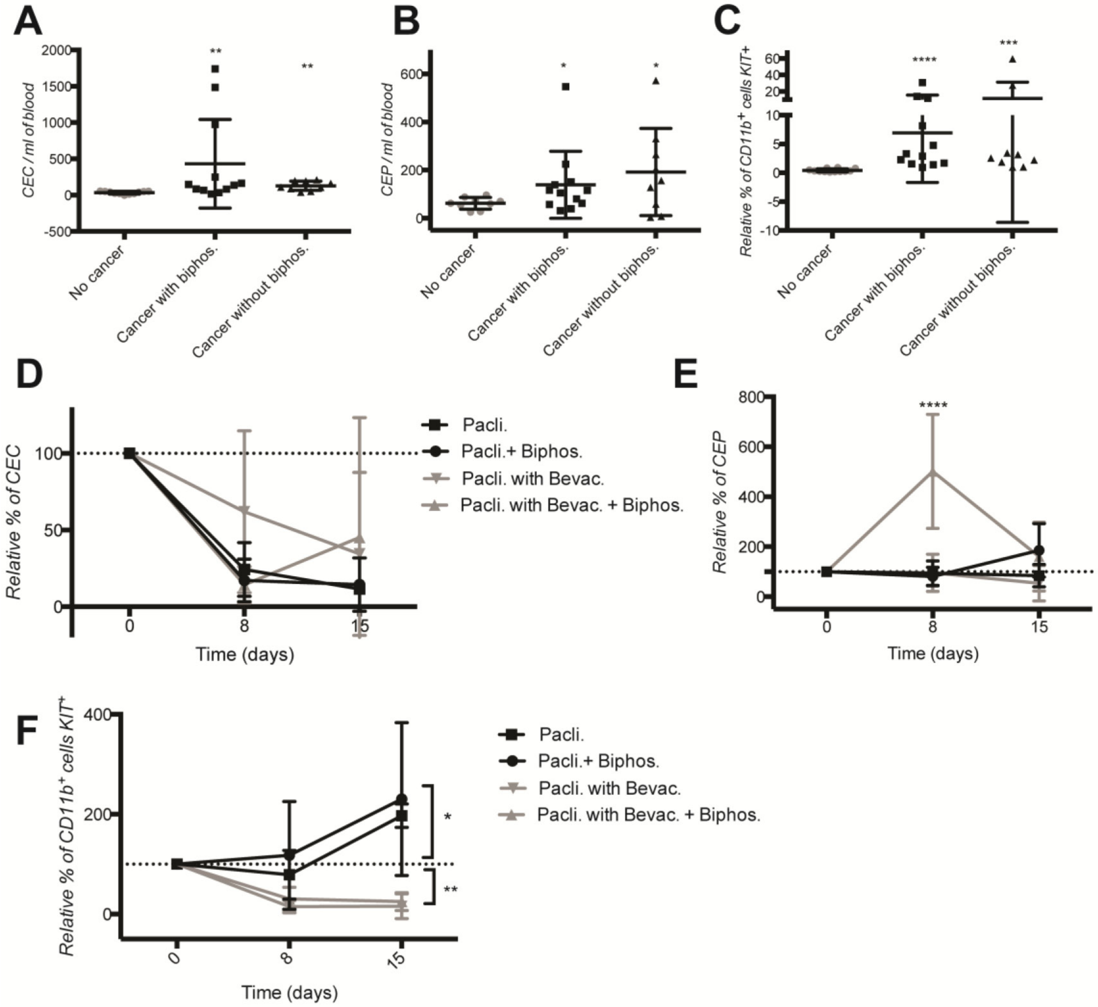

Supplementary Figure S5: Bisphosphonates impact on circulating cells. (**A**) Quantification of CEC and of (**B**) CEP in the blood of healthy donors and mBC patients ± bisphosphonate treatment as indicated. (**C**) Quantification of $CD11b^{+}$ cells expressing KIT in the blood of healthy donors and mBC patients ± bisphosphonate. (**D**) Relative level CEC and (**E**) CEP under both chemotherapy treatments ± bisphosphonate. (**F**) Relative levels CD11b+ cells expressing KIT under both chemotherapy treatments ± bisphosphonate. Cell quantifications were performed by flow cytometry. All data are represented as mean +/− SD.

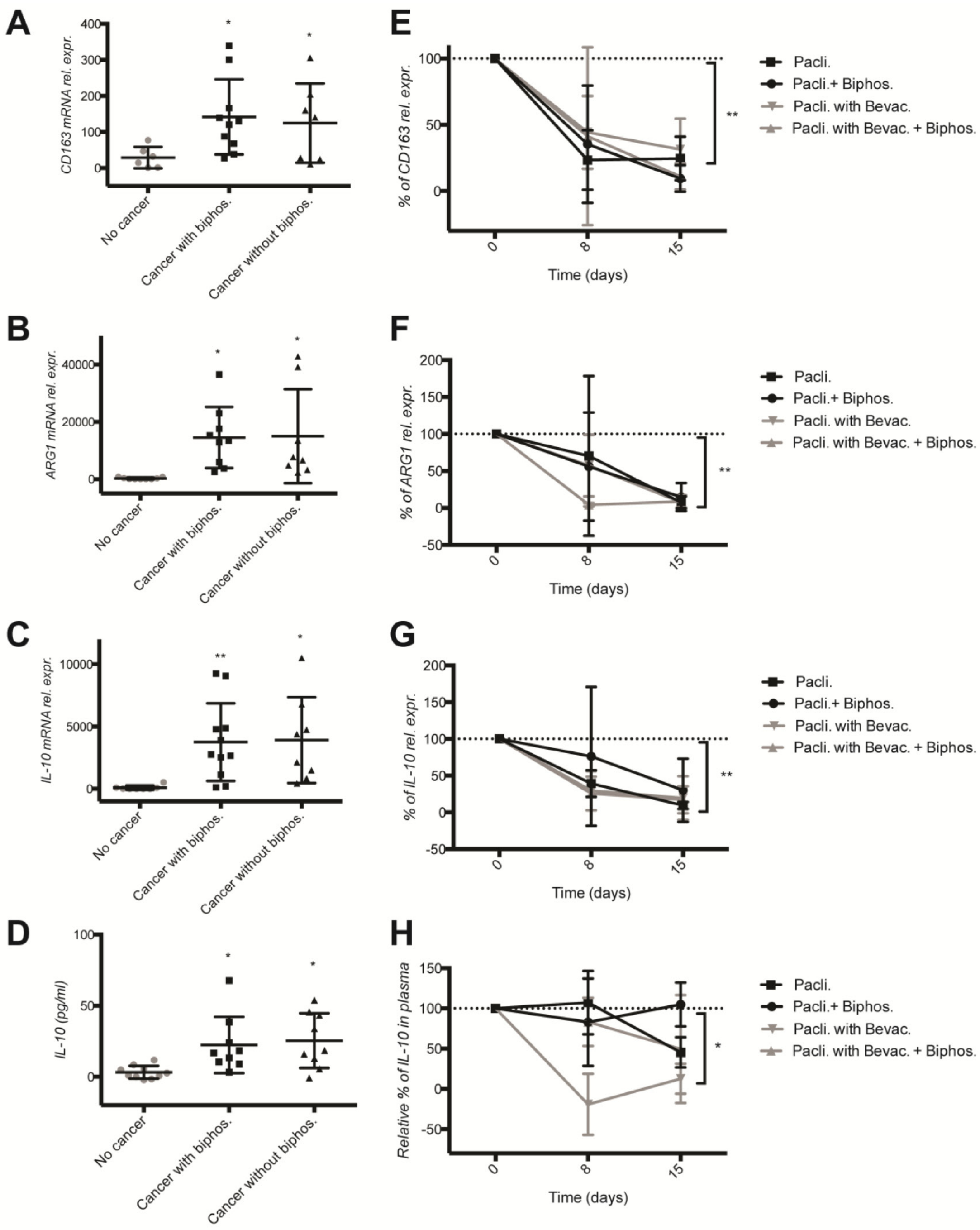

Supplementary Figure S6: Bisphosphonates impact on M2 markers. (**A**) Quantification of CD163, (**B**) ARG1 and (**C**) IL-10 mRNA expression level in CD11b+ cells of cancer patients ± bisphosphonate treatment. (**D**) Quantification of IL-10 in the plasma of cancer patients ± bisphosphonate treatment. (**E**) Relative changes in CD163, (**F**) ARG1 and (**G**) IL-10 mRNA expression level in CD11b+ cells of cancer patients under both chemotherapy treatments ± bisphosphonate. (**H**) Relative changes of IL-10 level in the plasma of cancer patients under both chemotherapy treatments ± bisphosphonate. mRNA quantifications were performed by real time qPCR and cytokines were measured by ELISA. All data are represented as mean +/− SD.

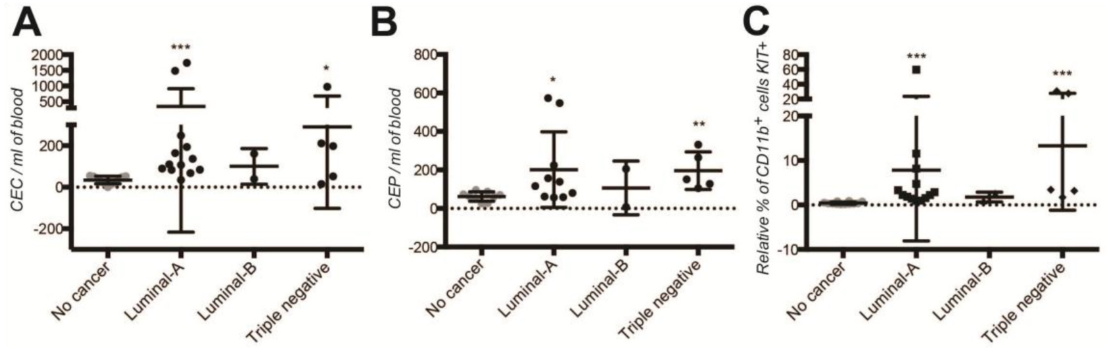

Supplementary Figure S7: Impact of breast cancer types on circulating cells. (**A**) Quantification of CEC in the blood of patient based of tumor type. (**B**) Quantification of CEP in the blood of patient based on tumor type. (**C**) Quantification CD11b^{+} monocytes expressing KIT based on tumor type. Cell quantifications were performed by flow cytometry. All data are represented as mean +/− SD.

Supplementary Table S1: List of significantly differentially expressed genes in patients vs heatly subjects determined by RNASeq

Supplementary Table S2: List of significantly up-regulated genes analysed by gene ontology

Supplementary Table S3: List of significantly down-regulated genes analysed by gene ontology
